# Multiomics Profiling Unveils Key Genes and Metabolites Involved in the Salt Tolerance of *Gossypium hirsutum*

**DOI:** 10.3390/genes17010022

**Published:** 2025-12-26

**Authors:** Zheng Weng, Fan Wang, Xin Wei, Lianjia Zhao, Wei Wang, Jianfeng Lei

**Affiliations:** 1 College of Agriculture, Xinjiang Agricultural University, 311 Nongda East Road, Urumqi 830052, China; 2 Institute of Crop Research, Xinjiang Uyghur Autonomous Region Academy of Agricultural Sciences, 403 Nanchang Road, Urumqi 830091, China; 3 Institute of Cotton Research, Xinjiang Uyghur Autonomous Region Academy of Agricultural Sciences, 403 Nanchang Road, Urumqi 830091, China

**Keywords:** *G. hirsutum*, salt stress, ABC transporter pathway, starch and sucrose metabolism

## Abstract

Background: Salt stress is a primary abiotic constraint on cotton growth, significantly impairing yield and fiber quality. Methods: To elucidate the regulatory mechanisms underlying salt stress responses in *Gossypium hirsutum*, we performed transcriptomic and metabolomic profiling at multiple time points following salt treatment. Results: We identified 33,975 differentially expressed genes (DEGs), with significant enrichment in pathways related to plant hormone signal transduction, amino acid metabolism, and starch and sucrose metabolism. K-means clustering grouped the DEGs into six expression modules corresponding to distinct response stages. Additionally, UPLC–MS analysis identified 6292 metabolites—spanning lipids, carbohydrates, and amino acids—and revealed substantial metabolic reprogramming with increasing stress duration. An integrated multiomics analysis highlighted the ABC transporter and starch and sucrose metabolism pathways as key regulatory modules for salt tolerance and identified critical genes within them. Conclusions: Collectively, these findings provide a comprehensive view of the transcriptional and metabolic dynamics of *G. hirsutum* under salt stress, offering valuable insights for understanding the molecular mechanisms of salt tolerance.

## 1. Introduction

*G. hirsutum* is among the world’s major economic crops, providing fiber for the textile industry and serving as an important oilseed and forage crop. However, soil salinization has become an increasingly serious issue and is one of the major abiotic stresses limiting cotton production [1]. It is estimated that 20% of the world’s arable land and 33% of irrigated farmland are affected by salinization, with this proportion continuing to increase at a rate of approximately 10% annually. If no effective measures are taken, more than 50% of the world’s arable land could be affected by salinity by 2050 [2]. Salt stress leads to a reduction in soil water potential, exacerbates ion toxicity, and causes nutrient imbalances, severely affecting seed germination, growth, and yield [3]. The effects of salt stress are particularly significant during the cotton germination and seedling stages, directly limiting plant growth, reducing yield, and impairing fiber quality [4]. Salt stress disrupts water absorption, ion balance, photosynthesis, and metabolic pathways, severely hindering normal growth and development [5]. Research has shown that salt stress not only reduces plant height, shortens root length, and decreases the number of lateral roots but also significantly affects dry matter accumulation, further impairing growth and development [6]. The imbalance of ions caused by salt stress is among the key factors leading to physiological dysfunction in cotton [7]. Specifically, salt stress decreases the K^+^ content and increases the Na^+^ content in leaves, which in turn disrupts cellular ion balance and membrane function [8]. Additionally, ion imbalance is accompanied by oxidative stress, with significant increases in malondialdehyde (MDA) and proline contents, further exacerbating cellular damage and inhibiting normal metabolic processes in cotton [9]. Therefore, elucidating the molecular response mechanisms of *G. hirsutum* to salt stress is highly important for revealing the genetic basis of salt tolerance, understanding the mechanisms of crop stress adaptation, and breeding salt-tolerant varieties.

With the rapid development of high-throughput sequencing and mass spectrometry technologies, integrated transcriptomic and metabolomic analyses have become powerful tools for revealing the mechanisms of plant stress responses [10]. Compared with single-omics analysis, multiomics integration allows for a system-level understanding of gene expression regulatory networks and metabolic reprogramming processes, enabling the identification of key regulatory genes, metabolites, and their interactions [11]. For example, in drought stress studies in sweet potato, a combined analysis of transcriptomic and metabolomic data revealed that drought-tolerant varieties resist drought by increasing amino acid and respiratory metabolism, increasing flavonoid accumulation, and activating the antioxidant system [12]. Further combined analysis indicated that drought responses involve mainly the regulation of oxidative balance, signal transduction, plant hormones, and energy metabolism. A joint analysis in rice revealed that pathways such as amino acid biosynthesis, starch and sucrose metabolism, and phenylpropanoid biosynthesis were most enriched, with amino acid biosynthesis having the strongest impact on salt tolerance [13]. In rice varieties with stronger salt tolerance, the transmembrane transport protein OsCCX2, amino acid transporter AAP3, and ABC transporter AB2E were significantly correlated with flavonoid metabolites, indicating that the coregulation of these transporters with secondary metabolism plays an important role in salt stress tolerance. Similarly, in wheat drought stress studies, multiomic analysis revealed that the drought-tolerant mutant T13 significantly upregulated the expression of genes related to flavonoid and phenolic acid biosynthesis (such as those related to *HCT*, *FLS*, *CHS*, and *F3′5′H*) under stress and resulted in the accumulation of corresponding metabolites. These metabolic pathways are considered key factors in the differential drought tolerance of wheat [14]. The results of this study also highlighted that drought stress leads to the production of large amounts of ROS and that plants maintain cellular homeostasis by increasing antioxidant enzyme activity and the accumulation of osmotic regulators, underscoring the importance of integrated analysis in revealing complex stress response mechanisms. In a study of alkaline stress in *G. hirsutum*, an integrative analysis of transcriptomics and metabolomics revealed flavonoid biosynthesis and alpha-linolenic acid metabolism as key regulatory pathways and selected a set of candidate genes associated with alkaline stress tolerance through weighted gene coexpression network analysis (WGCNA) [15]. These case studies demonstrate that multiomics integration not only reveals the complex networks of stress responses but also identifies key molecules and pathways that single-omics approaches often fail to capture. In particular, pathways related to ion transport, energy metabolism, antioxidants, and secondary metabolism repeatedly appear in response to abiotic stresses such as salt and drought, highlighting their core role in plant stress adaptation.

As a crucial global economic crop, *G. hirsutum* faces the growing challenge of salt stress. Analysis of the molecular responses of upland cotton under salt stress, particularly the relationship between gene expression and metabolite changes, is essential for understanding its salt tolerance mechanisms [16]. Although previous studies have conducted transcriptomic and metabolomic analyses of the response of cotton to salt stress, revealing mechanisms such as ion transport and antioxidant responses, a systematic analysis of the dynamic response process is lacking [6,17,18]. In particular, the transcriptional and metabolic changes and their associations between early and late stages of stress remain unclear. Furthermore, although existing studies have often focused on hormonal signals such as ABA or jasmonic acid, the roles of other pathways in salt stress have received insufficient attention. Therefore, in this study, the salt-tolerant upland cotton variety Xinluzao 17 (XLZ 17) was selected, and transcriptomic and metabolomic analyses were conducted at 0, 2, 4, 6, 12, 24, and 48 h under salt stress. The aim was to comprehensively elucidate the dynamic response mechanisms of salt tolerance from the perspectives of both gene expression and metabolite variation. Using integrated enrichment, time series clustering, and gene—metabolite correlation network analysis, we identified key genes and metabolites that may play central roles in salt tolerance. By performing a comprehensive analysis of salt stress responses at different time points, we aimed to identify critical genes, metabolites, and pathways related to salt tolerance, providing a scientific basis for further functional validation and varietal improvement. By deeply investigating the salt tolerance mechanisms in cotton, this study provides genetic and metabolic markers for the breeding of salt-tolerant varieties, thereby enhancing the productivity of cotton in saline-affected soil environments.

## 2. Materials and Methods

### 2.1. Plant Materials

Distinctness, uniformity, and stability (DUS) testing was first performed to confirm the genetic identity of the plant materials. Following this evaluation, the *G. hirsutum* cultivar Xinluzao 17 (XLZ 17), developed by the Early-Maturing Cotton Breeding Innovation Team of the Economic Crop Research Institute, Xinjiang Academy of Agricultural Sciences (Variety Approval No. Xinshenmian 2004-004), was selected for subsequent experiments. The variety exhibits good growth performance, high yield, and high fiber quality and is salt tolerant. The experiments were conducted in the indoor laboratory of the College of Agriculture, Xinjiang Agricultural University, from November–December 2024. Uniform, plump seeds were surface-sterilized with 75% ethanol and soaked in distilled water at 28 °C in the dark for 12 h. After radicle emergence, the seeds were sown into seedling trays containing a 1:1 (*v*/*v*) mixture of nutrient soil and vermiculite. The seedlings were subsequently grown at 28 °C under a 16 h light/8 h dark photoperiod until the second true leaf was fully expanded.

Uniformly growing seedlings were gently removed from the substrate, rinsed thoroughly with distilled water, and transferred to beakers containing a 200 mmol/L NaCl solution for salt stress treatment. Leaf samples were harvested at 0, 2, 4, 6, 12, 24, and 48 h posttreatment, with three biological replicates per time point. Immediately after sampling, the leaves were wrapped in aluminum foil, snap-frozen in liquid nitrogen, and stored at −80 °C until further analysis.

### 2.2. RNA-Seq Library Construction and Sequencing

Samples were shipped on dry ice to Biomarker Technologies (Beijing, China) for RNA-seq analysis. Total RNA was extracted using TRIzol reagent (Invitrogen, Waltham, MA, USA) and fragmented prior to cDNA synthesis. First-strand cDNA was synthesized using random primers, followed by second-strand synthesis. The resulting cDNA fragments were end-repaired, A-tailed, and ligated with sequencing adapters, followed by PCR enrichment to generate the final libraries. Library quality was assessed using an Agilent 2100 Bioanalyzer (Agilent, CA, USA) and qPCR.

High-throughput sequencing was performed on the Illumina HiSeq 2500 platform. Raw reads were processed using fastp to remove adapter sequences, low-quality reads, and reads containing >5% ambiguous bases (N), yielding clean reads for downstream analysis [19]. These clean reads were aligned to the *G. hirsutum* TM-1 reference genome (ZJU-AD1_v2.1) using HISAT2 [20]. Gene abundance was quantified using feature counts, and differential expression analysis was conducted with DESeq2 [21]. Differentially expressed genes (DEGs) were identified based on the criteria of |fold change| ≥ 2 and false discovery rate (FDR) < 0.01. FDR was employed to control the multiple comparison problem to ensure the reliability of the screening results. Finally, GO and KEGG enrichment analyses were performed using the clusterProfiler package [22].

### 2.3. Metabolite Extraction

Freeze-dried leaf samples were ground into a fine powder, and 50 mg was extracted with 1000 μL of extraction solvent (methanol–acetonitrile–water, 2:2:1, *v*/*v*/*v*). The samples were vortexed for 30 s, homogenized at 45 Hz for 10 min, and sonicated for 10 min in an ice-water bath. Following incubation at −20 °C for 1 h, the extracts were centrifuged at 12,000 rpm for 15 min. A 500 μL aliquot of the supernatant was collected and dried in a vacuum concentrator. The dried residue was reconstituted in 160 μL of acetonitrile–water (1:1, *v*/*v*), vortexed for 30 s, sonicated for 10 min, and centrifuged again at 12,000 rpm for 15 min. The final supernatant was used for LC–MS analysis.

Metabolomic analysis was performed using a Waters Acquity I-Class PLUS UPLC system coupled to an AB Sciex QTRAP 6500+ mass spectrometer (SCIEX, Boston, MA, USA). Chromatographic separation was carried out on an Acquity UPLC HSS T3 column (1.8 μm, 2.1 × 100 mm) with an injection volume of 2 μL. The electrospray ionization (ESI) source parameters were set as follows: source temperature, 550 °C; ion spray voltage, +5500 V (positive mode) and −4500 V (negative mode); ion source gas I, gas II, and curtain gas pressures were 50, 55, and 35 psi, respectively; and collision-induced dissociation (CID) was set to medium.

### 2.4. Metabolomic Data Processing and Analysis

Metabolites were identified using Progenesis QI software (version 2.0) by matching MS/MS spectra against the METLIN database, other public repositories, and Biomarker’s in-house database. Prior to analysis, redundant signals, including isotopic peaks, adducts (containing K^+^, Na^+^ or NH_4_^+^), and fragment ions, were removed. Quantitative analysis was subsequently performed using the multiple reaction monitoring (MRM) mode in a triple quadrupole mass spectrometer.

The peak areas were normalized across all the samples prior to statistical analysis. Principal component analysis (PCA) was performed in R to visualize metabolic variation among the samples [23]. Metabolites were classified and mapped to biochemical pathways using KEGG annotation [24]. Additionally, partial least squares regression (PLSR) models were constructed to evaluate the relationships between metabolite profiles and sample categories. Differentially regulated metabolites (DRMs) were identified based on a fold change > 2 or <0.5 and a *p* value < 0.05 [25]. DRMs also needed to meet the criterion of VIP (VIP > 1), with the VIP value extracted from the OPLS-DA results. The OPLS-DA results also included score plots and permutation plots generated using the R package MetaboAnalystR (version 4.0). The data were subjected to Log_2_ transformation and mean centering before OPLS-DA was performed. To avoid overfitting, 200 permutation tests were conducted. These stringent criteria ensured the biological significance of the selected DRMs, providing a robust foundation for the downstream interpretation of metabolic responses.

### 2.5. Integrated Transcriptomic and Metabolomic Analyses

KEGG enrichment analysis of DEGs and DRMs was performed using the ClusterProfiler package in R [22]. By comparing the enriched pathways in the transcriptome and metabolome, KEGG pathways that were commonly enriched in both the transcriptome and the metabolome were identified. For the commonly enriched KEGG pathways, the changes in gene and metabolite contents within these pathways were further displayed. The heatmap package in R was used to generate heatmaps to show the expression patterns of related genes and metabolites across all the samples. Correlation analysis was conducted using the quantitative values of genes and metabolites across all the samples. The Pearson correlation coefficient was calculated to assess the correlation between each DEG and DRM. Gene–metabolite pairs with correlation coefficients greater than 0.80 and *p* values less than 0.05 were selected for network construction and visualization.

### 2.6. qRT–PCR Analysis

Total RNA was extracted using an E.Z.N.A. Plant RNA Kit (Omega Bio-Tek, Doraville, GA, USA). RNA concentration and purity were assessed using a NanoDrop 2000 spectrophotometer (Thermo Fisher Scientific, Waltham, MA, USA). First-strand cDNA was synthesized from 1 μg of total RNA using a PrimeScript RT Reagent Kit with gDNA Eraser (Takara Bio Inc., Shiga, Japan). qRT–PCR was performed on a Roche LightCycler 480 system using SYBR Green Master Mix (Takara Bio Inc.). The amplification protocol consisted of initial denaturation at 95 °C for 30 s, followed by 40 cycles of 95 °C for 5 s and 60 °C for 34 s. Relative gene expression was calculated using geNorm, with *GhUBQ7* as the internal reference gene [26]. Three biological replicates were performed for each sample, and all primer sequences are listed in Table S1.

## 3. Results

### 3.1. Global Analysis of RNA-Seq Data

**Figure 1:**
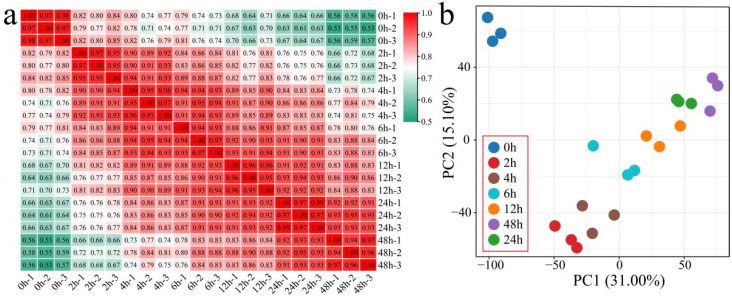
Correlation and PCA of RNA-seq samples. (**a**) Correlation coefficients among samples, (**b**) PCA of all samples.

To investigate the transcriptional response to salt stress, 21 samples of XLZ 17 were collected across seven time points (0, 2, 4, 6, 12, 24, and 48 h) for RNA-seq analysis. After filtering, a total of 128.73 Gb of clean data were obtained, with each sample yielding at least 5.74 Gb. The percentage of bases with Q30 quality scores exceeded 96.70%, and the mapping rate to the TM-1 reference genome was consistently above 94.51% (Table S2). Reproducibility was confirmed using Pearson’s correlation analysis, with all biological replicates displaying coefficients greater than 0.94 (Figure 1a). Furthermore, PCA revealed that biological replicates clustered tightly, whereas samples from different stress durations were clearly separated from the control (0 h) (Figure 1b). Collectively, these results demonstrate the high quality and consistency of the transcriptome dataset, validating its suitability for downstream analysis.

### 3.2. Differential Expression Analysis

**Figure 2:**
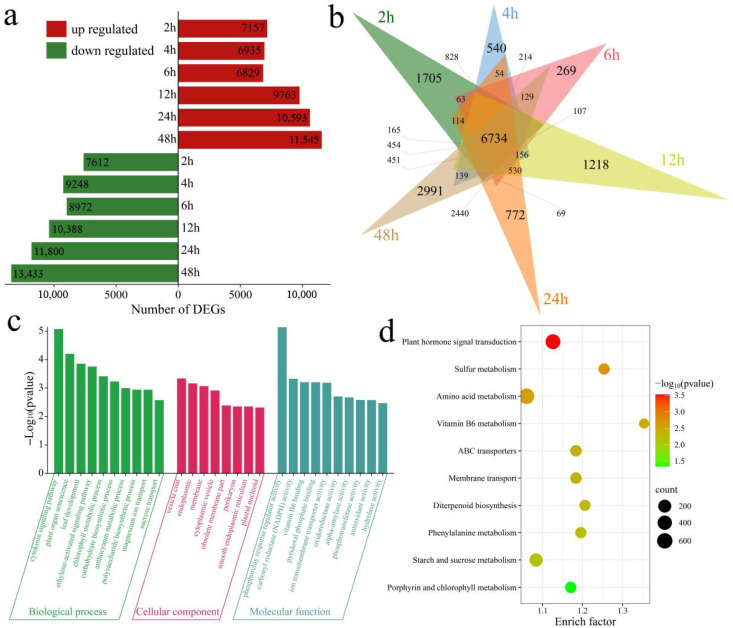
Identification and enrichment analysis of DEGs. (**a**) Numbers of up- and downregulated DEGs at each time point, (**b**) Venn diagram of shared and unique DEGs, (**c**) GO enrichment, (**d**) KEGG enrichment.

Differential expression analysis revealed a total of 33,975 DEGs, with 6734 shared across all time points (Figure 2a,b). Compared with the control (0 h), 14,769 DEGs were identified at 2 h (7157 upregulated and 7612 downregulated), of which 1705 were unique to this stage. The number of DEGs increased slightly at 4 h to 16,183 (540 unique) and remained stable at 6 h, with 15,801 DEGs (269 unique). As the duration of stress increased, the transcriptional response intensified: 20,151 DEGs were identified at 12 h (1218 unique), and 22,393 were identified at 24 h (772 unique). By 48 h, the number of DEGs peaked at 24,978 (11,545 upregulated and 13,433 downregulated), which included 2991 unique genes. GO enrichment analysis of the 33,975 DEGs revealed significant involvement in cytokinin and ethylene-activated signaling; plant organ senescence; leaf development; signaling and development; metabolism; chlorophyll, carbohydrate, anthocyanin, and polysaccharide biosynthesis; and magnesium ion and sucrose transport (Figure 2c). KEGG pathway analysis revealed significant overrepresentation of transport processes, plant hormone signal transduction, ABC transporters, membrane transport, and multiple metabolic pathways, including the starch and sucrose metabolism and porphyrin and chlorophyll metabolism pathways (Figure 2d).

### 3.3. Clustering Analysis of DEGs

**Figure 3:**
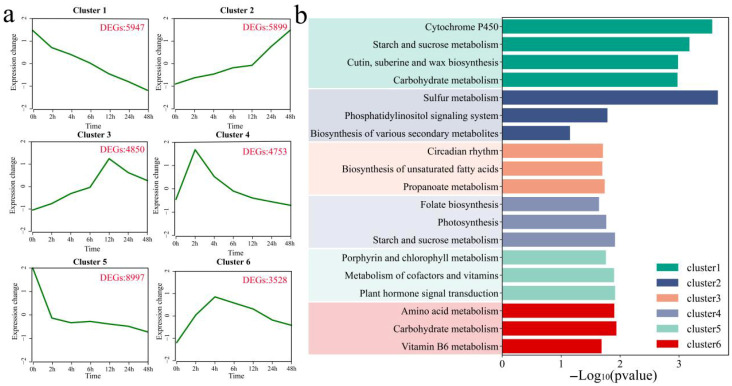
Clustering of DEGs and KEGG enrichment. (**a**) Line plots of expression patterns, (**b**) KEGG enrichment for each cluster.

K-means clustering was used to identify six statistically significant clusters from 33,975 DEGs, and KEGG pathway enrichment analysis was performed on each cluster (Figure 3a,b). The expression of Cluster 1 decreased after stress, reaching its lowest level at 48 h, and included 5947 DEGs. Significant annotations were found for the cytochrome P450, starch and sucrose metabolism, cutin, suberin and wax biosynthesis, and carbohydrate metabolism pathways. The expression of Cluster 2 increased after stress, reaching its highest level at 48 h, and included 5899 DEGs. Significant enrichment was detected for the sulfur metabolism, phosphatidylinositol signaling system, and biosynthesis of various secondary metabolite pathways. The expression of Cluster 3 increased after stress, reaching its maximum at 12 h but then decreasing, and included 4850 DEGs. Significant annotations were found in the circadian rhythm, biosynthesis of unsaturated fatty acids, and propanoate metabolism pathways. Cluster 4, containing 4753 DEGs, showed an increase in expression levels 2 h after stress, followed by a decrease. It was significantly annotated in the folate biosynthesis, photosynthesis, and starch and sucrose metabolism pathways. Cluster 5, containing 8997 DEGs, showed a decrease in expression levels 2 h after stress, followed by gradual stabilization. Porphyrin and chlorophyll metabolism, metabolism of cofactors and vitamins, and plant hormone signal transduction pathways were significantly annotated. Cluster 6, containing 3528 DEGs, showed a maximum increase in expression levels 4 h after stress, followed by a gradual decrease. It was significantly annotated in the amino acid metabolism, carbohydrate metabolism, and vitamin B6 metabolism pathways.

### 3.4. Metabolomic Analysis

**Figure 4:**
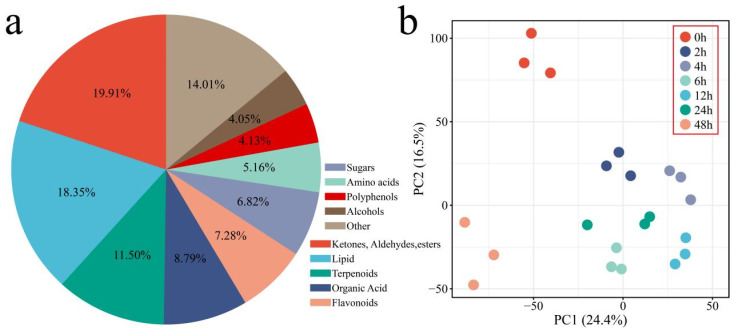
Classification and PCA of metabolomic profiles. (**a**) Percentage distribution of metabolite classes, (**b**) PCA of metabolomic samples.

UPLC–MS revealed a total of 6292 metabolites from 21 samples collected across seven time points (0, 2, 4, 6, 12, 24, and 48 h) under salt stress. These metabolites were classified into major categories, primarily including ketones, aldehydes, and esters (19.91%), lipids (18.35%), terpenoids (11.5%), organic acids (8.79%), flavonoids (7.28%), sugars (6.82%), amino acids (5.16%), polyphenols (4.13%), and alcohols (4.05%) (Figure 4a). Principal component analysis (PCA) confirmed the reliability and reproducibility of the metabolomics data. The biological replicates clustered tightly, whereas the salt-stressed samples were clearly distinct from the control (0 h), revealing significant metabolic shifts. These results indicate that the cotton metabolome undergoes dynamic, time-dependent changes in response to salt stress, confirming the high quality of the dataset for further analysis (Figure 4b).

### 3.5. Identification of DRMs

**Figure 5:**
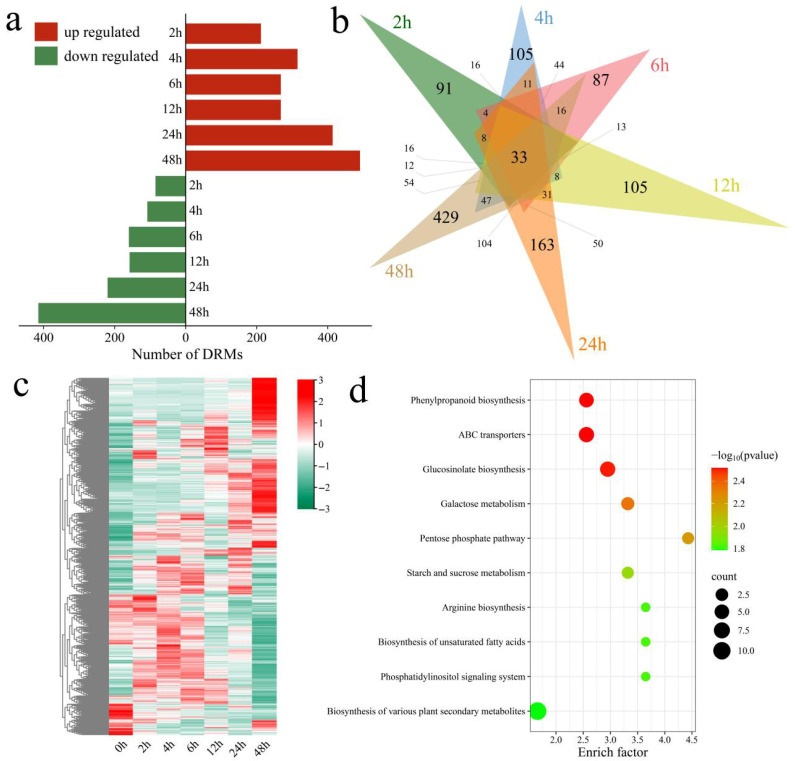
DRM identification and enrichment analysis. (**a**) Up- and downregulated DRMs, (**b**) Shared and unique DRMs, (**c**) Heatmap of DRM clustering; (**d**) KEGG enrichment of DRMs.

Differential analysis revealed a total of 1756 DRMs, 33 of which were shared across all treatment groups (Figure 5a,b). Compared with the control (0 h), 297 DRMs were identified at 2 h (212 upregulated, 85 downregulated), 91 of which were unique to this stage. At 4 h, the number of DRMs increased to 423 (315 upregulated, 108 downregulated), including 105 unique metabolites. At 8 h, 428 DRMs were detected (268 upregulated and 160 downregulated), 87 of which were unique. At 12 h, 426 DRMs were identified (268 upregulated and 158 downregulated), 105 of which were unique. The number of DRMs increased to 634 at 24 h (414 upregulated, 220 downregulated; 163 unique). After 48 h, the response peaked, with 906 DRMs identified (491 upregulated and 415 downregulated), including 429 unique metabolites. Cluster analysis of the 1756 DRMs revealed that metabolite abundance shifted significantly with stress duration, reflecting the dynamic regulatory mechanisms governing the cotton salt stress response (Figure 5c). During the early stage (2 h), the metabolite levels remained relatively stable and exhibited only minor fluctuations. As salt stress persisted (6 h and beyond), the number of metabolites whose abundance changed markedly increased. Notably, at later time points (12, 24, and 48 h), the levels of most metabolites increased significantly, indicating that cotton plants undergo active metabolic reprogramming under prolonged salt stress. KEGG enrichment analysis revealed significant enrichment in pathways such as phenylpropanoid biosynthesis, ABC transporters, glucosinolate biosynthesis, galactose metabolism, the pentose phosphate pathway, starch and sucrose metabolism, arginine biosynthesis, biosynthesis of unsaturated fatty acids, the phosphatidylinositol signaling system, and biosynthesis of various plant secondary metabolites (Figure 5d).

### 3.6. Integrated Transcriptomic and Metabolomic Analyses

**Figure 6:**
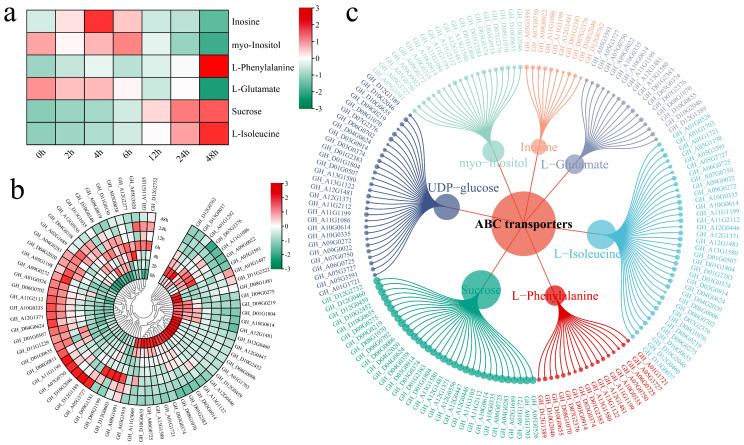
Integrated analysis of the ABC transporter pathway. (**a**) Metabolite heatmap, (**b**) Gene expression heatmap, (**c**) Gene–metabolite correlation network.

Integrated KEGG enrichment analysis of DEGs and DAMs revealed significant coenrichment in the ABC transporter and starch and sucrose metabolism pathways (Figure 2d and Figure 5d). Consequently, we prioritized the analysis of metabolite changes, gene expression patterns, and gene–metabolite correlations within the ABC transporter pathway (Figure 6). The abundances of major metabolites associated with this pathway—such as inositol, L-phenylalanine, L-glutamate, sucrose, and L-isoleucine—increased significantly with prolonged salt stress (Figure 6a). Notably, at 24 and 48 h poststress, the accumulation of specific metabolites (such as inositol and L-glutamate) was significant, suggesting that these compounds play a critical role in the salt stress response. Concurrently, genes associated with the ABC transporter pathway began to exhibit differential expression trends in the early stages (2 and 4 h), with the magnitude of these changes peaking at 48 h (Figure 6b). Specifically, the expression levels of multiple genes increased significantly at 24 and 48 h. To further investigate the regulatory relationship between ABC transporter genes and metabolites, we performed a correlation analysis. Gene–metabolite pairs exhibiting strong correlations (absolute correlation coefficient > 0.8, *p* < 0.05) were selected for visualization (Figure 6c). L-Isoleucine and sucrose exhibited the greatest degree of connectivity with ABC transporter genes; specifically, L-isoleucine was significantly correlated with 35 genes, and sucrose was linked to 33 genes. Furthermore, seven specific genes (*GH_A12G1481*, *GH_D01G2383*, *GH_A10G0335*, *GH_A13G1580*, *GH_D03G0374*, *GH_D10G0655*, and *GH_D08G1070*) were significantly associated with the seven top-ranked metabolites.

**Figure 7:**
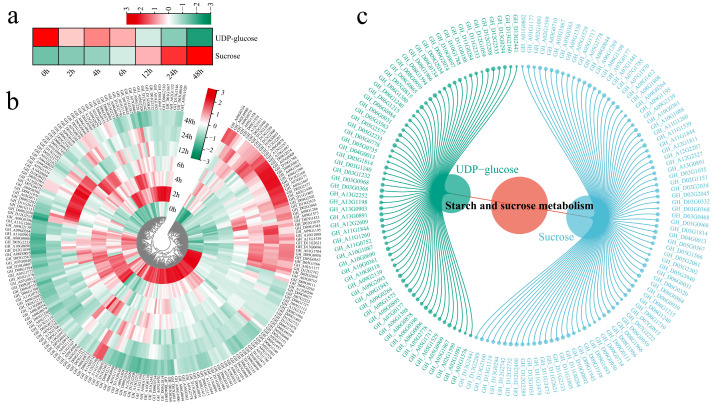
Integrated analysis of starch and sucrose metabolism. (**a**) Metabolite heatmap, (**b**) Gene expression heatmap, (**c**) Correlation network.

We further analyzed metabolite accumulation, gene expression patterns, and gene–metabolite correlations within the starch and sucrose metabolism pathways (Figure 7). The abundance of UDP-glucose decreased slightly during the early stages of stress (0–2 h), reaching its lowest levels at 24 and 48 h. In contrast, the abundance of sucrose tended to decrease, peaking at 48 h (Figure 7a). The expression levels of genes associated with starch and sucrose metabolism were most significantly upregulated at 24 and 48 h, suggesting that these genes play pivotal roles in the salt stress response in cotton (Figure 7b). We calculated correlations between genes and metabolites within the starch and sucrose metabolism pathway, visualizing pairs with absolute correlation coefficients (∣r∣) > 0.8 and *p* < 0.05 (Figure 7c). Most genes were significantly associated with UDP-glucose and sucrose, suggesting that these genes play a synergistic role in regulating the synthesis and transport of these key metabolites during the salt stress response.

### 3.7. qRT–PCR Validation of RNA–Seq Data

**Figure 8:**
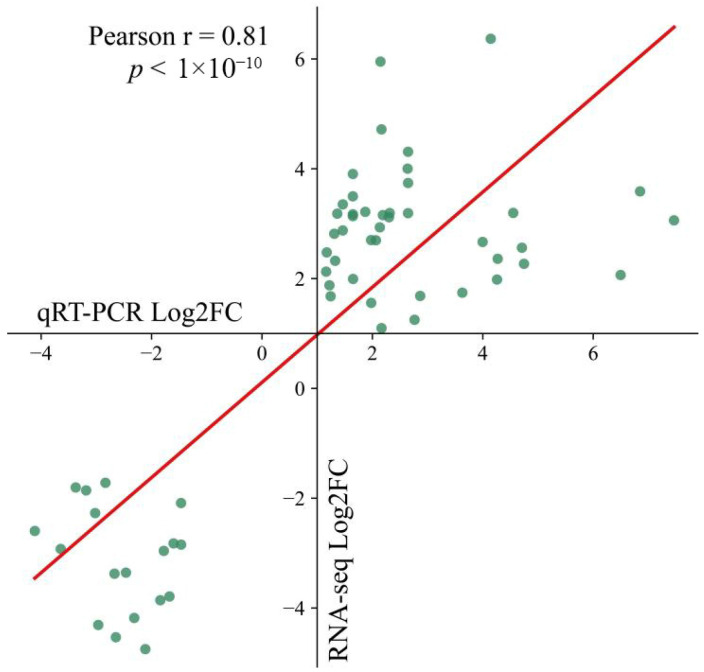
Correlations between the qRT–PCR and RNA-seq expression data.

To validate the accuracy of the RNA-seq analysis, we randomly selected 10 genes for qRT–PCR verification. The qRT–PCR expression profiles were strongly correlated with the RNA-seq data (r = 0.81, *p* < 0.05), confirming the consistency and reliability of the transcriptomic dataset. These results provide a robust foundation for subsequent research into the functions of genes that respond to salt stress (Figure 8).

## 4. Discussion

Cotton is a globally significant economic crop that serves as the primary raw material for the textile industry and is a valuable oilseed resource [27]. However, the rapid expansion of soil salinization worldwide has become a major constraint on cotton production [2]. Estimates indicate that more than 20% of irrigated agricultural land is currently affected by salinity, a proportion projected to increase due to climate change, excessive water use, and improper irrigation practices [28]. Under salt stress, plants encounter multiple physiological challenges, including ion toxicity (characterized by excessive Na^+^ accumulation and K^+^ loss), osmotic stress, and secondary oxidative damage [29]. During the early stages of salt exposure, plants rapidly activate ion transport systems, such as SOS1 and members of the NHX family, to extrude Na^+^, retain K^+^, and maintain cellular ion homeostasis [30]. Concurrently, reactive oxygen species (ROS) levels increase sharply, necessitating the activation of antioxidant defense pathways to mitigate oxidative injury [31,32]. For example, in barley, significant transcriptional changes in *SOS1/2/3* and *NHX1/2/3* were observed within 24 h of salt treatment, whereas SOD, POD, and CAT activities strongly correlated with Na^+^/K^+^ flux patterns [33,34,35]. Additionally, compatible solutes such as proline and branched-chain amino acids contribute to early stress mitigation by stabilizing proteins, maintaining osmotic balance, and preserving cellular structural integrity [36]. These responses are frequently accompanied by Ca^2+^ signaling waves and MAPK cascade activation, which regulate transcription factors such as MYB, WRKY, and NAC, thereby orchestrating downstream ion transport and stress-response networks [37]. Consistent with these mechanisms, cluster analysis of the DEGs in the present study revealed that early salt-responsive genes (2 and 4 h) in cotton were enriched primarily in pathways related to signal transduction, antioxidative responses, and ion homeostasis. At later stages (24 and 48 h), extensive metabolic reprogramming became more pronounced, suggesting a transition from acute stress responses to long-term acclimation. This shift is consistent with observations in other crops, where sugars, amino acids, flavonoids, and fatty acid derivatives accumulate substantially as stress persists [35,38].

Previous studies have integrated transcriptomic and metabolomic analyses of cotton under salt or alkaline stress, with the majority focusing on ABA or jasmonic acid signaling pathways [18,39,40,41]. However, this study detected significant enrichment in the cytokinin signaling pathway, ethylene-activated signaling pathway, organ senescence, and leaf development processes, revealing new roles for growth-regulating hormones in the salt stress response. Furthermore, the study identified relevant receptors and regulatory genes, which have rarely been reported in the literature. Although these studies emphasize the crucial role of secondary metabolite biosynthesis pathways, this research focuses specifically on anthocyanin metabolism, carbohydrate and polysaccharide biosynthesis, and sucrose transport pathways, further revealing the potential roles of anthocyanins and polysaccharides in osmoregulation and antioxidant responses [42]. Previous research has focused mainly on the transport mechanisms of Na^+^, K^+^, and Cl^−^, whereas this study detected significant enrichment in magnesium ion transport and sucrose transport, indicating that Mg^2+^ balance and sugar redistribution are important components of cotton’s salt tolerance [41,43]. KEGG analysis also revealed significant enrichments in sulfur metabolism and vitamin B_6_ metabolism, which have rarely been reported in previous cotton salt stress studies. As a cofactor in the antioxidant system, vitamin B_6_ suggests that new metabolic pathways are involved in the salt stress response. Additionally, this study detected significant enrichment in ATP-binding cassette (ABC) transporters, membrane transport, diterpene biosynthesis, and phenylalanine metabolism pathways, indicating the important roles of transmembrane transport and terpenoid/phenolic secondary metabolites in salt stress.

ABC transporters play a pivotal role in salt tolerance by mediating the ATP-dependent translocation of ions, hormones, lipids, and secondary metabolites, thereby maintaining ion homeostasis, hormonal balance, and ROS regulation [44]. The differential regulation of ABC transporters under salt stress has been documented in numerous species. For example, the overexpression of *AtABCG36* enhances Na^+^ efflux, reduces leaf chlorosis, and promotes biomass accumulation under salt and drought conditions [45]. For instance, *OsRST31* modulates cytokinin transport from roots to shoots and acts as a negative regulator of salt tolerance [46]. In contrast, *ABC1K10A* positively regulates salt tolerance through the modulation of ROS [47]. In Arabidopsis, the *ABCG17* and *ABCG18* proteins function as ABA importers, mediating long-distance ABA transport from shoots to roots. Their downregulation under salt stress reduces ABA uptake, thereby facilitating ABA redistribution to maintain the balance between growth and stress responses [48]. *ZmMRPA6* enhances salt and cold tolerance in maize, and its homologs, *AtABCC4* and *AtABCC14*, confer similar salt-tolerant phenotypes [49]. In line with these findings, our integrated transcriptomic and metabolomic analyses demonstrated that the ABC transporter pathway plays a pivotal role in cotton salt tolerance. Specifically, L-isoleucine and sucrose were significantly correlated with 35 and 33 ABC transporter genes, respectively. Furthermore, we identified seven core genes (*GH_A12G1481*, *GH_D01G2383*, *GH_A10G0335*, *GH_A13G1580*, *GH_D03G0374*, *GH_D10G0655*, and *GH_D08G1070*) that were strongly associated with all key pathway metabolites, implicating them as central regulators in the salt-adaptation network of *G. hirsutum*. Notably, although we detected strong correlations between genes and metabolites, these correlations do not prove a functional regulatory relationship. Correlation analysis provides potential biological clues; however, further experimental validation is needed to confirm the regulatory mechanisms between these gene–metabolite pairs.

Carbohydrate metabolism is fundamental to both plant development and stress responses [50]. Sucrose, glucose, fructose, and other soluble sugars derived from starch degradation contribute to osmotic adjustment, turgor maintenance, and the provision of energy for cellular repair [51]. When photosynthesis is compromised under salt stress, starch mobilization and sucrose resynthesis become essential for sustaining basal metabolic activity and fueling the production of compatible solutes, such as proline [52]. In addition to their metabolic roles, sugars directly stabilize proteins and membranes and serve as signaling molecules that coordinate stress responses via crosstalk with hormone pathways [53]. In cotton, for instance, the downregulation of the methyltransferase gene *GhDMT7* under salt stress leads to reduced genome-wide DNA methylation (particularly of the CHH type), which subsequently activates key genes involved in starch and sucrose metabolism [54]. Similarly, carbohydrate metabolism is strongly activated in the halophyte Suaeda following salt exposure [55]. Consistent with these observations, the starch and sucrose metabolism pathway was significantly enriched in our study, with most genes positively correlated with glucose and sucrose accumulation. These findings suggest that modulating carbohydrate metabolism is a primary strategy employed by *G. hirsutum* to maintain osmotic balance and energy supply under saline conditions. In this study, only a single variety was used for salt stress research, and a comprehensive comparison of salt-tolerant and salt-sensitive varieties is lacking, which is indeed a limitation of this study. Future research should consider including more varieties with distinct salt tolerance differences for a comprehensive assessment of the differences in response among various genotypes under salt stress, thereby providing a better understanding of the genetic basis of salt tolerance mechanisms. Moreover, different cotton varieties exhibit varying performance under salt stress; thus, further research is needed to validate these key pathways and genes in more salt-tolerant or salt-sensitive materials, with the aim of further confirming and expanding the findings of this study. Future studies should prioritize the functional validation of key ABC transporter and starch/sucrose metabolism genes, potentially utilizing advanced genetic approaches such as CRISPR-based genome editing to enhance these traits. Furthermore, given that comprehensive resistance evaluation integrates field phenotyping, laboratory assays, and molecular analyses, our findings provide valuable reference data for DUS testing and the assessment of salt–alkali tolerance in new cotton cultivars.

## 5. Conclusions

Through integrated transcriptomic and metabolomic analyses of XLZ 17 at different time points under salt stress, the dynamic response pattern of *G. hirsutum* from early rapid stress to later metabolic reprogramming was systematically characterized. The results highlight the core roles of ABC transporters and the starch and sucrose metabolism pathways in the process of salt tolerance. Moreover, multiple significantly associated metabolites and key genes were identified, providing important molecular clues for understanding the salt stress response in cotton. Future research needs to further analyze the precise biosynthetic pathways and upstream and downstream regulatory networks of metabolites in these pathways, identify the core regulatory genes involved in their biosynthesis and transport processes, and further clarify the roles of these metabolites and genes in cotton salt tolerance. Through gene editing, marker-assisted breeding, and physiological regulation methods, cotton yield and fiber quality can be improved under saline–alkaline conditions, promoting the sustainable development of the cotton industry on less fertile land.

## Data Availability

We have deposited the raw data into the GSA database of the National Genomics Data Center (NGDC) with the accession number PRJCA052352. The original contributions presented in this study are included in the article/Supplementary Material. Further inquiries can be directed to the corresponding authors.

## Supplementary Materials

The following supporting information can be downloaded at: https://www.mdpi.com/article/10.3390/genes17010022/s1, Table S1: All primers used in this study; Table S2: Summary statistics for RNA-seq analysis of *G. hirsutum* under Salt.
